# Network-Based Logistic Classification with an Enhanced *L*_1/2_ Solver Reveals Biomarker and Subnetwork Signatures for Diagnosing Lung Cancer

**DOI:** 10.1155/2015/713953

**Published:** 2015-06-16

**Authors:** Hai-Hui Huang, Yong Liang, Xiao-Ying Liu

**Affiliations:** Faculty of Information Technology & State Key Laboratory of Quality Research in Chinese Medicines, Macau University of Science and Technology, Avenida Wai Long, Taipa 999078, Macau

## Abstract

Identifying biomarker and signaling pathway is a critical step in genomic studies, in which the regularization method is a widely used feature extraction approach. However, most of the regularizers are based on *L*_1_-norm and their results are not good enough for sparsity and interpretation and are asymptotically biased, especially in genomic research. Recently, we gained a large amount of molecular interaction information about the disease-related biological processes and gathered them through various databases, which focused on many aspects of biological systems. In this paper, we use an enhanced *L*_1/2_ penalized solver to penalize network-constrained logistic regression model called an enhanced *L*_1/2_ net, where the predictors are based on gene-expression data with biologic network knowledge. Extensive simulation studies showed that our proposed approach outperforms *L*_1_ regularization, the old *L*_1/2_ penalized solver, and the Elastic net approaches in terms of classification accuracy and stability. Furthermore, we applied our method for lung cancer data analysis and found that our method achieves higher predictive accuracy than *L*_1_ regularization, the old *L*_1/2_ penalized solver, and the Elastic net approaches, while fewer but informative biomarkers and pathways are selected.

## 1. Introduction

Identifying molecular biomarker or signaling pathway involved in a phenotype is a particularly important problem in genomic studies. Logistic regression is a powerful discriminating method and has an explicit statistical interpretation which can obtain probabilities of classification regarding the class label information.

A key challenge in identifying diagnosis or prognosis biomarkers using the logistic regression model is that the number of observations is much smaller than the size of measured biomarkers in most of the genomic studies. Such limitation causes instability in the algorithms used to select gene marker. Regularization methods have been widely used in order to deal with this problem of high dimensionality. For example, Shevade and Keerthi proposed the sparse logistic regression based on the Lasso regularization [1, 2]. Meier et al. investigated logistic regression with group Lasso [3]. Usually, the Lasso type procedures are often called *L*_1_-norm type regularization methods. However, *L*_1_ regularization may yield inconsistent selections when applied to variable selection in some situations [4] and often introduces the extra bias in the estimation [5]. In many genomic studies, we need a sparser solution for interpretation and accurate outcomes, but *L*_1_ regularization has a gap to meet these requirements. Thus, a further improvement of regularization is urgently required. *L*_*q*_ (0 < *q* < 1) regularization can assuredly generate more sparse and precise solutions than *L*_1_ regularization. Moreover, *L*_1/2_ penalty can be taken as a representative of *L*_*q*_ (0 < *q* < 1) penalty and has demonstrated many attractive properties which do not appear in some *L*_1_ regularization approaches, such as unbiasedness, sparsity, and oracle properties [6–8].

So far, we observed dense molecular interaction information about the disease-related biological processes and gathered it through databases focused on many aspects of biological systems. For example, BioGRID records collected various biological interactions from more than 43,468 publications [9]. These regulatory relationships are usually represented by a network. Combining these pieces of graphic information extracted from the biological process with an analysis of the gene-expression data had provided useful prior information to detective noise and removes confounding factors from biological data for several classification and regression models [10–14].

Inspired by the aforementioned methods and ideas, here, we define a network-constrained logistic regression model with *L*_1/2_ penalty following the framework established by [11], where the predictors are based on the gene-expression data with biologic network knowledge. The proposed model is aimed at identifying some biomarkers and subnetworks regarding diseases. In order to achieve a better prediction, we use an enhanced half thresholding algorithm for *L*_1/2_  regularization, which is more efficient than the old half thresholding approach in the literature [6, 15, 16].

The rest of the paper is organized as follows. In Section 2, we proposed a new version of the network-constrained logistic regression model with *L*_1/2_ regularization. In Section 3, we presented an enhanced half thresholding method for *L*_1/2_ regularization and the corresponding coordinate descent algorithm. In Section 4, we evaluated the performance of our proposed approach on the simulated data and presented the applications of the proposed methods to an analysis of lung cancer data. We concluded the paper with Section 5.

## 2. *L*_1/2_ Penalized Network-Constrained Logistic Regression Model

Generally, assuming that dataset *D* has *n* samples, *D* = {(*X*_1_, *y*_1_), (*X*_2_, *y*_2_),…, (*X*_*n*_, *y*_*n*_)}, where *X*_*i*_ = (*x*_*i*1_, *x*_*i*2_,…, *x*_*ip*_) is *i*th sample with *p* genes and *y*_*i*_ is the corresponding variable that takes a value of 0 or 1. Define a classifier *f*(*x*) = *e* ^*x*^/(1 + *e* ^*x*^) and the logistic regression is defined as

(1) $$\begin{array}{c} P\left(\;y_{i}=1\;|\;X_{i}\;\right)=f\left(\;{X^{\prime }}_{i}\beta \;\right)=\frac{\exp\left({X^{\prime }}_{i}\beta \right)}{1+\exp\left({X^{\prime }}_{i}\beta \right)}, \end{array}$$

where *β* = (*β*_1_,…, *β*_*p*_) are the coefficients to be estimated. We can obtain *β* by minimizing the log-likelihood function of the logistic regression. Following [11], to combine biological network with an analysis of the gene microarray data, we used a Laplacian constraint approach here. Consider a graph *G* = (*V*, *E*), where *V* is the set of genes that meet *p* explanatory variables and *E* is the set of edges. If gene *u* and gene *v* are connected, then there is an edge between gene *u* and gene *v*, which is denoted by *E*_*uv*_ = 1; else *E*_*uv*_ = 0. *w*_*uv*_ denotes the weight of edge *E*_*uv*_. The normalized Laplacian matrix *L* for *G* is defined by

(2) $$\begin{array}{c} L_{uv}=\left\{\begin{array}{cc} 1-\frac{w_{uv}}{d_{u}} & \text{if }u=v,\;\;d_{u}\neq 0,\\ -\frac{w_{uv}}{\sqrt{d_{u}d_{v}}} & \text{if }u,v\text{ are adjacent}\\ 0 & \text{otherwise}, \end{array}\right. \end{array}$$

where *d*_*u*_ and *d*_*v*_ are the degrees of genes *u* and *v*, respectively. The degrees of gene *u* (or *v*) describe the number of the edges that connected with *u* (or *v*). For *λ* ≥ 0, the network-constrained logistic regression model is presented as

(3) Lλ,β=−∑i=1nyilog⁡fX′iβ+1−yilog⁡1−fX′iβ+λβTLβ,

where the first term in (3) is the log-likelihood function of the logistic model and the second term is a network constraint based on the Laplacian matrix, which induces a smooth solution of *β* on the graph.

Directly computing (3) performs poorly for both prediction and biomarker selection purposes when the gene number *p* ≫ the sample size *n*. Therefore, the regularization approach is vitally needed. When adding a regularization term to (3), the sparse network-constrained logistic regression can be written as

(4) Lλ1,λ2,β=−∑i=1nyilog⁡fX′iβ+1−yilog⁡1−fX′iβ+λ1∑j=1pPβj+λ2βTLβ,

where *λ*_1_ > 0 is a regularization parameter. In Zhang et al. [13], the authors used Lasso (*L*_1_) which has the regularization term *P*(*β*) = ∑_*j*=1_^*p*^|*β*_*j*_| to penalize (4). However, the result of the Lasso type (*L*_1_) regularization is not good enough for interpretation, especially in genomic research. Besides this, *L*_1_ regularization is asymptotically biased [17, 18]. To improve the solution's sparsity and its predictive accuracy, we need to think beyond *L*_1_ regularization to *L*_*q*_ penalties. In mathematics, *L*_*q*_  (0 < *q* < 1) type regularization |*β*|_*q*_ = ∑|*β*|^*q*^ with the lower value of *q* would lead to better solutions with more sparsity and gives asymptotically unbiased estimates [17]. Moreover, *L*_1/2_ penalty can be taken as a representative of *L*_*q*_  (0 < *q* < 1) penalty and has permitted an analytically expressive thresholding representation [6, 7]. Therefore, we proposed a novel *L*_1/2_ net approach based on *L*_1/2_ regularization to penalize the network-constrained logistic regression model, as shown in

(5) Lλ1,λ2,β=−∑i=1nyilog⁡fX′iβ+1−yilog⁡1−fX′iβ+λ1β1/2+λ2βTLβ,

where |*β*|_1/2_ = ∑_*j*=1_^*p*^|*β*_*j*_|^1/2^.

## 3. A Coordinate Descent Algorithm for the Network-Constrained Logistic Model with the Enhanced *L*_1/2_ Thresholding Operator

*L*_1/2_ penalty function is nonconvex, which raises numerical challenges in fitting the models. Recently, the coordinate descent algorithms [19] for solving nonconvex regularization models (SCAD [20], MCP [21]) have shown significant efficiency and convergence [22]. Since the computational burden increases only linearly with the feature number *p*, the coordinate descent algorithm can be a powerful tool for solving high-dimensional problems. Its standard procedure can be demonstrated as follows: for every coefficient *β*_*j*_  (*j* = 1,2,…, *p*), to partially optimize the target function with respect to *β*_*j*_, and fix the remaining elements *β*_*k*_(*k* = 1,2,…, *p*  and  *k* ≠ *j*) at their most recently updated values. The specific form of updating *β* depends on the thresholding operator of the penalty.

In this paper, we present an enhanced *L*_1/2_ thresholding operator for the coordinate descent algorithm:

(6) βj=Enhanced_Halfωj,λ=23ωj1+cos⁡2π−φλωj3if  ωj>5434λ2/30otherwise,

where *φ*_*λ*_(*ω*) = arccos⁡((*λ*/8)(|*ω*|/3)^−3/2^), *π* = 3.14, $\omega_{j}=\sum_{i=1}^{n}x_{ij}\left(y_{i}-{{\tilde{y}}_{i}}^{\left(j\right)}\right)$, and ${{\tilde{y}}_{i}}^{\left(j\right)}=\sum_{k\neq j}x_{ik}^{}\beta_{k}$ as the partial residual for fitting *β*_*j*_.

*Remark*. This enhanced *L*_1/2_ thresholding operator $(\sqrt[{3}]{54}/4){\left(\lambda \right)}^{2/3}$ outperforms the old *L*_1/2_ thresholding (3/4)(*λ*)^2/3^ introduced in [6, 15, 16]. We know that the quantity of the regularization solutions depends seriously on the value of the regularization parameter *λ*. Based on this enhanced *L*_1/2_ thresholding operator, when *λ* is chosen by some efficient strategies for the parameter tuning, such as cross validation, the convergence of algorithm (6) is proved [7].

The Laplacian matrix *L* is nonnegative definite; thus, it can be written as *L* = *SS* ^*T*^ by Cholesky decomposition. Following C. Li and H. Li [11] approach, (4) can be expressed as

(7) Lλ1,λ2,β=Lγ,β∗=−∑i=1nyi∗log⁡fX∗iβ∗+1−yi∗log⁡1−fX∗iβ∗+∑j=1pγβj∗1/2,

where ${X^{*}}_{(n+p)\times p}={\left(1+\lambda_{2}\right)}^{-1/2}\left(\begin{array}{c} X\\ \sqrt{\lambda_{2}}S^{T} \end{array}\right)$, $Y_{\left(n+p\right)}^{*}=\left(\begin{array}{c} Y\\ 0 \end{array}\right)$, $\beta^{*}=\sqrt{1+\lambda_{2}}\beta$, and *γ* is the regularization parameter and can be expressed as $\gamma =\lambda_{1}/\sqrt{1+\lambda_{2}}$.

One-term Taylor series expansion for (7) can be written as

(8) Lγ,β∗≈12n∑i=1nZi−X∗iβ∗′WiZi−X∗iβ∗+∑j=1pPβ∗j,

where $Z_{i}={X^{*}}_{i}\tilde{\beta^{*}}+\left(y_{i}^{*}-f\left({X^{*}}_{i}\tilde{\beta^{*}}\right)\right)/f\left({X^{*}}_{i}\tilde{\beta^{*}}\right)(1-f({X^{*}}_{i}\tilde{\beta^{*}})$ is the estimated response and $W_{i}=f({X^{*}}_{i}\tilde{\beta^{*}})(1-f({X^{*}}_{i}\tilde{\beta^{*}})$ is the weight for the estimated response. $f\left({X^{*}}_{i}\tilde{\beta^{*}}\right)=\exp\left({X^{*}}_{i}\tilde{\beta^{*}}\right)/(1+\exp\left({X^{*}}_{i}\tilde{\beta^{*}}\right))$ is the evaluated value under the current parameters. Thus, we can redefine the partial residual for fitting current $\tilde{\beta^{*}}$ as ${\overset{ˇ}{Z}}_{i}^{\left(j\right)}=\sum_{i}^{n}W_{i}({\tilde{Z}}_{i}-\sum_{k\neq j}x_{ik}^{*}{\tilde{\beta^{*}}}_{k})$ and $\omega_{j}=\sum_{i=1}^{n}x_{ij}^{*}(Z_{i}-{\overset{ˇ}{Z}}_{i}^{\left(j\right)})$. The procedure of the coordinate descent algorithm for *L*_1/2_ penalized network-constrained logistic model is described as follows.

**Algorithm 1 (the coordinate descent algorithm for *L*_1/2_ penalized network-constrained logistic model).**

We consider the following.

*Step 1*. Initialize all *β*_*j*_(*m*) ← 0 (*j* = 1,2,…, *p*) and *y* ^*∗*^, *X* ^*∗*^, and set *m* ← 0, *γ* chosen by cross validation.

*Step 2*. Calculate *Z*(*m*) and *W*(*m*) and approximate the loss function (8) based on the current *β*(*m*).

*Step 3*. Update each *β*_*j*_(*m*) and cycle over *j* = 1,…, *p*, until *β*_*j*_(*m*) does not change.

*Step 3.1*. Compute *Z*_*i*_^(*j*)^(*m*) ← ∑_*i*=1_^*n*^ *W*_*i*_(*m*)(*Z*_*i*_(*m*) − ∑_*k*≠*j*_ *x*_*ik*_^*∗*^ *β*_*k*_(*m*)) and $\omega_{j}\left(m\right)\leftarrow \sum_{i=1}^{n}x_{ij}(Z_{i}(m)-{{\overset{ˇ}{Z}}_{i}}^{\left(j\right)}\left(m\right))$.

*Step 3.2*. Update *β*_*j*_(*m*) ← Enhanced_Half(*ω*_*j*_(*m*), *γ*).

*Step 4*. Let *m* ← *m* + 1, *β*(*m* + 1) ← *β*(*m*).

If *β*(*m*) dose not converge, then repeat Steps 2 and 3.

## 4. Simulation and Application

### 4.1. Analyses of Simulated Data

We evaluate the performance of four methods: the network-constrained logistic regression models with *L*_1_ regularization (*L*_1_ net), *L*_1/2_ regularization with old thresholding value (3/4)(*λ*)^2/3^ (*L*_1/2_ net) and with the enhanced thresholding value $(\sqrt[{3}]{54}/4){\left(\lambda \right)}^{2/3}$ (enhanced *L*_1/2_ net), and the Elastic net regularization approach (Elastic net). We first simulated the graph structure to mimic gene regulatory network: assuming that the graph consists of 200 independent transcription factors (TFs) and each TF regulates 10 unlike genes, so there are a total of 2200 variables, *X* = (*x*_1_, *x*_2_,…, *x*_*p*_), *p* = 2200. The training and the independent test data sets include the sample sizes of 100, respectively. Each TF *x*_*n*_ and its regulated genes *x*_*m*_ were generated by the normal distribution *N*(0,1). We set the correlation rate between *x*_*n*_ and its regulated gene *x*_*m*_ as 0.75, *x*_*m*_ = (1 − 0.75) × *x*_*m*_ + (0.75) × *x*_*n*_. The binary responder *y*_*i*_ (1 ≤ *i* ≤ 100), which is associated with the matrix *X* of TFs and their regulated genes, is calculated based on the following formula and rule:

(9) yi=1 Label  1,if  Pyi=1 ∣ Xi=exp⁡Xiβ+ϵ1+exp⁡Xiβ+ϵ⩾0.5;  else  yi=0 Label  0,

where $\beta =(2,\underset{10}{\underset{︸}{2/\sqrt{5},\ldots ,2/\sqrt{5}}},-2,\underset{10}{\underset{︸}{-2/\sqrt{5},\ldots ,-2/\sqrt{5}}},4$, $\underset{10}{\underset{︸}{4/\sqrt{5},\ldots ,4/\sqrt{5}}},-4,\underset{10}{\underset{︸}{-4/\sqrt{5},\ldots ,-4/\sqrt{5}}},0,\ldots ,0)$ for Model 1, and *ε* ~ *N*(0, *σ*_*e*_^2^).

Model 2 was defined similar to Model 1, except that we considered the case when the TF can have positive and negative effects on its regulated genes at the same time:

(10) β=2,−25,−25,−25,25,…,25︸7,−2,25,25,25,−25,…,−25︸7,4,−45,−45,−45,45,…,45︸7,4,45,45,45,−45,…,−45︸7,0,…,0.

In these two models, the 10-fold cross validation approach was conducted on the training datasets to tune the regularization parameters of the enhanced *L*_1/2_ net, *L*_1/2_ net, and *L*_1_ net. Both penalized parameters for *L*_1_ and ridge regularization in the Elastic net were tuned by the 10-fold cross validation on the two-dimensional parameter surfaces. We repeated the simulations over 100 times and then computed the misclassification error, the sensitivity, and the specificity averagely for each net model on the test datasets.

Table 1 summarizes the simulation results from each regularization net model. In general, our proposed enhanced *L*_1/2_ net model achieved the smallest misclassification errors in Models 1 (9.22%) and 2 (10.76%) compared with the other regularization methods including the old *L*_1/2_ thresholding method (9.85% for Model 1 and 10.83% for Model 2), *L*_1_ net (11.81% for Model 1 and 13.21% for Model 2), and the Elastic net (13.12% for Model 1 and 14.14% for Model 2). Meanwhile, the enhanced *L*_1/2_ net resulted in the highest sensitivity in Model 1 (98.5%) compared with the other methods. Moreover, the enhanced *L*_1/2_ net obtained the best specificity in Model 2 (98.7%) amongst the other approaches. To sum up, the enhanced *L*_1/2_ net outperforms the other three algorithms in terms of prediction accuracy, sensitivity, and specificity.

### 4.2. Analysis of Lung Cancer

In this section, we merged the protein-protein interaction (PPI) network (see http://thebiogrid.org/) with a lung cancer (LC) gene-expression dataset [23] to demonstrate the performance of our proposed enhanced *L*_1/2_ net method. The gene-expression dataset contains the expression profiles of 22284 genes for 107 patients, in which 58 had lung cancer. To test the generalization ability of the proposed method, we divided the dataset into the training set (sample size *n* = 70; 38 LC, 32 non-LC) which covered 2/3 samples of the dataset and the test set (sample size *n* = 37; 20 LC, 17 non-LC) which covered the other 1/3 specimens of the dataset. The 10-fold cross validation approach was conducted on the training dataset to tune the regularization parameters. By combining the gene-expression data with the PPI network, the final PPI network includes 8619 genes and 28293 edges.

**Figure 1:**
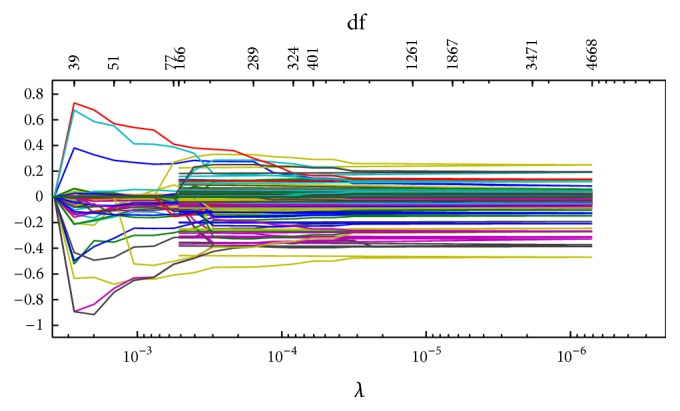
The solution paths of the enhanced *L*_1/2_  net for the lung cancer dataset in one sample run.

**Figure 2 fig2:**
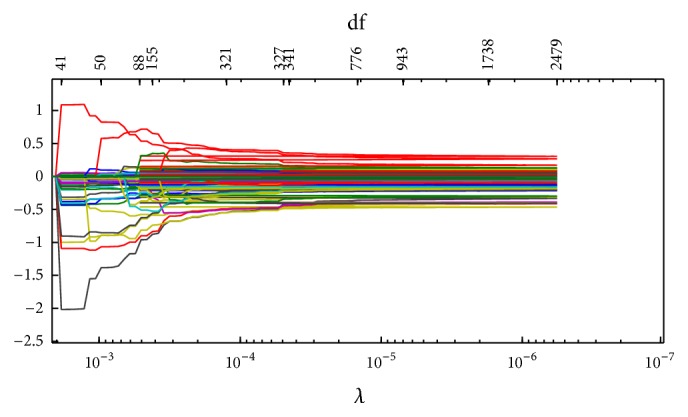
The solution paths of *L*_1/2_ net for the lung cancer dataset in one sample run.

**Figure 3 fig3:**
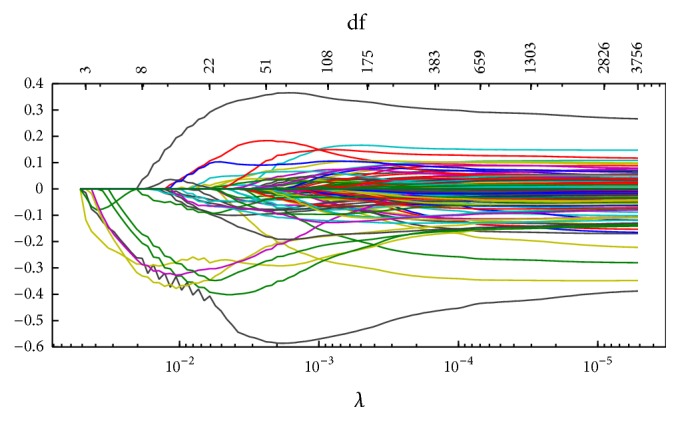
The solution paths of *L*_1_ net for the lung cancer dataset in one sample run.

**Figure 4:**
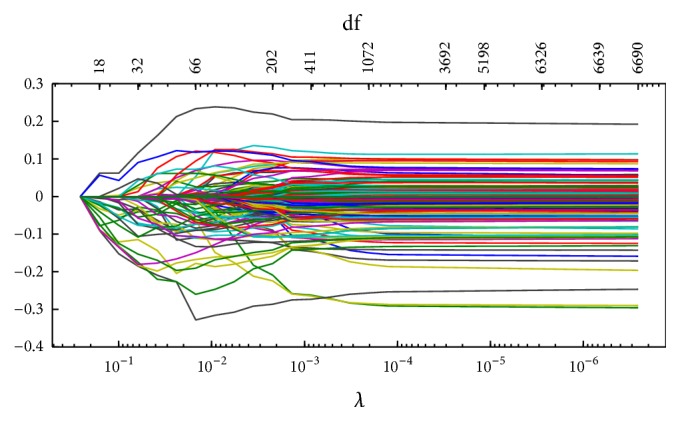
The solution paths of the Elastic net for the lung cancer dataset in one sample run.

Figures 1 [Fig fig2] [Fig fig3]–4 display the solution paths of the four regularization net methods for the LC dataset in one sample run. Here, *x*-axis displays the values of the running lambda (the running lambda of *L*_1_ penalty in the Elastic net approach), and *x*-axis at the top (degrees of freedom) means the number of nonzero coefficients of beta. *y*-axis is the values of the coefficients beta which measure the gene importance. The predictive model builds from the training set and then tests its predictive performance on the test set. The detailed results were represented in Table 2.

As shown in Table 2, the enhanced *L*_1/2_ net selected the fewest number of genes and edges compared to *L*_1/2_ net, *L*_1_ net, and the Elastic net. Meanwhile, the predictive performance of the enhanced *L*_1/2_ net outperforms the other three regularization net algorithms.

**Figure 5:**
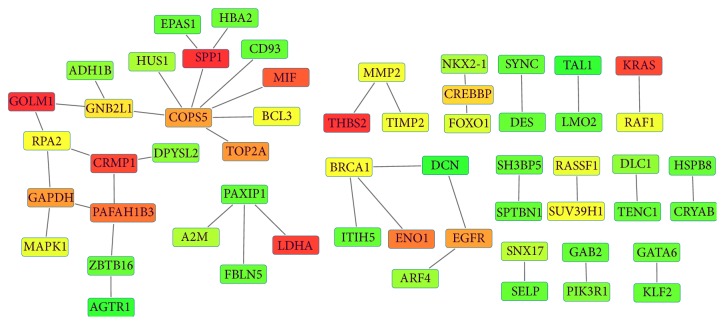
Subnetworks identified by the enhanced *L*_1/2_ net for lung cancer datasets (only those genes that are linked on the PPI network are plotted).

To further evaluate the performance of the enhanced *L*_1/2_ net procedure, we report its capacity of identifying the biomarkers related to lung cancer. NK2 homeobox 1 (Nkx2-1) protein regulates transcription of genes specific for lung. It is used as a biomarker to determine lung cancer in anatomic pathology. It also has a critical role in maintaining lung tumor cells [24, 25]. Epidermal growth factor receptor (EGFR) is known to play a key role in cell proliferation and apoptosis. EGFR overexpression and activity could result in tumor growth and progression [26] and somatic mutations within the tyrosine kinase domain of EGFR, which have been identified in a subset of lung adenocarcinoma [27, 28]. The enhanced *L*_1/2_ net (Figure 5) and *L*_1/2_ net successfully identified these two important biomarkers for LC. However, neither *L*_1_ net nor the Elastic net selected them both.

Except to identify these two significant biomarkers (EGFR and Nkx2-1), the enhanced *L*_1/2_ net also selected several pathways that were associated with lung cancer. For example, one of the subnetworks includes genes involving molecular proliferation (e.g., genes ARF4, EGFR, DCN, BRCA1, and ITIH5). As these gene express significantly and continuously, it promotes lung cancer progression. On the other hand, this group is linked to ENO1. We are unable to get a clear testimony to sustain this relationship by looking at PPI database. However, a recent report [29] has demonstrated that ENO1 is the promising biomarker that may provide more diagnostic efficacy for lung cancer. This link implies a functional relationship and suggests the important role of ENO1 in lung cancer.

All these results reveal that the enhanced *L*_1/2_ net is more reliable than *L*_1/2_ net, *L*_1_ net, and the Elastic net approaches for selecting key markers from high-dimensional genomic data. Another advantage of our proposed method is that it has the ability to recognize novel and potential relationships with biologic significance. It is mentionable that our proposed method is inclined to identify fewer but more informative genes (or edges) than *L*_1_ net and the Elastic net approaches in genomic data and that means the proposed method has allowed the researcher to more easily concentrate on the key targets for functional studies or downstream applications.

## 5. Conclusions

In biological molecular research, especially for cancer, the analysis of combining biological pathway information with gene-expression data may play an important role to search for new targets for drug design. In this paper, we use the enhanced *L*_1/2_ solver to penalized network-constrained logistic regression model to integrate lung cancer gene-expression with protein-to-protein interaction network. We develop the corresponding coordinate descent algorithm as a novel biomarker selection approach. This algorithm is extremely fast and easy to implement. Both simulation and real genomic data studies showed that the enhanced *L*_1/2_ net is a ranking procedure compared with *L*_1/2_ net (using the old thresholding operator), *L*_1_ net, and the Elastic net in the selection of biomarker and subnetwork.

We successfully identified several important clinical biomarkers and subnetwork that are driving lung cancer. The proposed method has provided new information to investigators in biological studies and can be the efficient tool for identifying cancer related biomarker and subnetwork.

## Supplementary Material

“Sub-networks identified by the L_1 net and the Elastic net for lung cancer datasets (only those genes that are linked on the PPI network are plotted). Nodes colored based on higher (red) to lower (green) coefficients in the model.”

## Figures and Tables

**Table 1:** Simulation results of the enhanced *L*_1/2_ net, *L*_1/2_ net, *L*_1_ net, and Elastic net, respectively.

Model	Misclassification errors (%)				Sensitivity (%)				Specificity (%)			
	Eh_*L*_1/2_	*L*_1/2_	*L*_1_	Elastic	Eh_*L*_1/2_	*L*_1/2_	*L*_1_	Elastic	Eh_*L*_1/2_	*L*_1/2_	*L*_1_	Elastic
1	**9.22**	9.85	11.81	13.12	**0.985**	0.971	0.968	0.873	0.969	0.970	0.962	0.981
	(0.36)	(0.31)	(0.41)	(0.12)	(0.00)	(0.00)	(0.02)	(0.00)	(0.00)	(0.01)	(0.01)	(0.00)

2	**10.76**	10.83	13.21	14.14	0.939	0.939	0.943	0.835	**0.987**	0.981	0.987	0.980
	(0.33)	(0.36)	(0.24)	(0.23)	(0.00)	(0.00)	(0.01)	(0.00)	(0.02)	(0.01)	(0.01)	(0.00)

Simulation results (averaged over 100 runs) for comparison of misclassification errors, sensitivity, and specificity used the enhanced *L*_1/2_ net, *L*_1/2_ net, *L*_1_ net, and the Elastic net, respectively. The standard errors are given in parentheses.

**Table 2:** The results of the enhanced *L*_1/2_ net, *L*_1/2_ net, *L*_1_ net, and Elastic net on LC dataset, respectively.

	Selected genes	Connected genes	Connected edges	Cross validation error	Test error
Eh_*L*_1/2_ net	171	54	41	6/70	5/37
*L*_1/2_ net	193	61	47	6/70	6/37
*L*_1_ net	500	150	121	7/70	6/37
Elastic	636	337	510	6/70	6/37

Results of analysis of LC gene expression dataset by four procedures, including the number of genes selected, the number of linked PPI network genes, the number of linked PPI network edges, the CV error, and test errors.
