# Supplementary material for: Network-Based Logistic Classification with an Enhanced L 1/2 Solver Reveals Biomarker and Subnetwork Signatures for Diagnosing Lung Cancer
Source: Biomed Res Int. 2015 Jun 16;2015:713953. doi: 10.1155/2015/713953 (PMC4488258; doi:10.1155/2015/713953)
Supplement: Supplementary file 1 — “Sub-networks identified by the L_1 net and the Elastic net for lung cancer datasets (only those genes that are linked on the PPI network are plotted). Nodes colored based on higher (red) to lower (green) coefficients in the model.” [file 713953.f1.zip › Additional File 1.pdf]

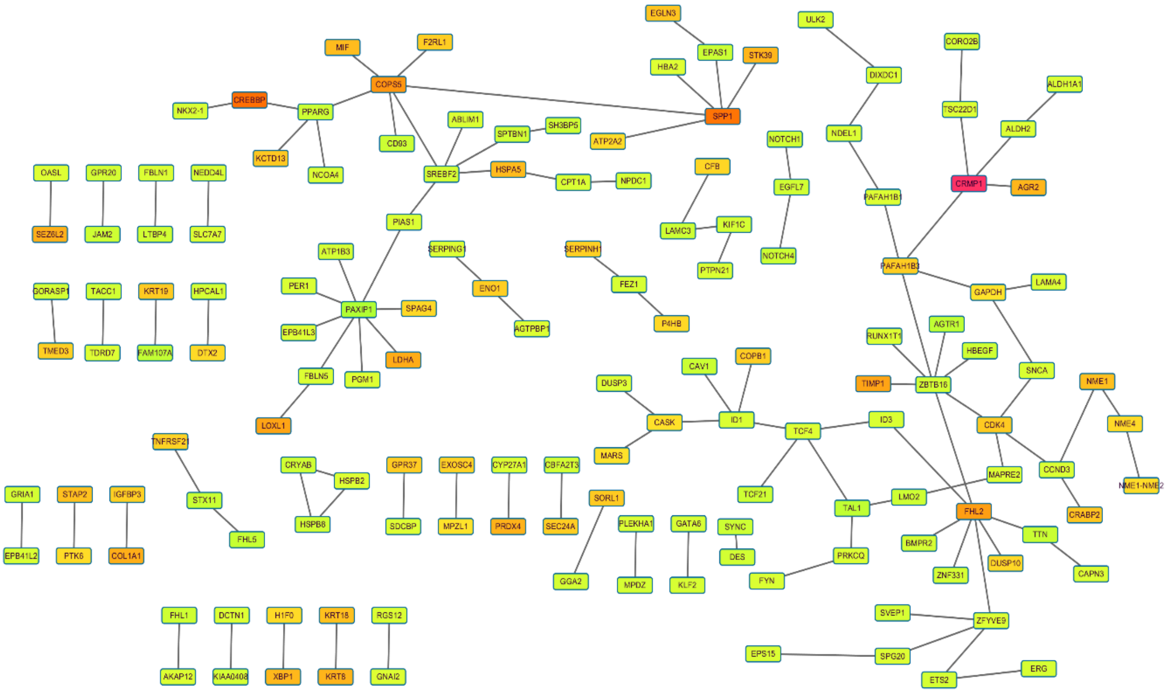

Additional File 1. Subnetworks identified by L_1 net for lung cancer datasets (only those genes that are linked on the PPI network are plotted).
